# Memory deficit following resection of an intraventricular myxoid glioneuronal tumor impinging on the bilateral fornix: A case report

**DOI:** 10.3389/fonc.2023.1263556

**Published:** 2023-09-27

**Authors:** Alena Stasenko, Erik Kaestner, Jonathan Rodriguez, Jiwandeep S. Kohli, Nikdokht Farid, Vanessa Goodwill, Marc S. Schwartz, Jessica D. Schulte, Carrie R. McDonald

**Affiliations:** ^1^ Center for Multimodal Imaging and Genetics, University of California San Diego, San Diego, CA, United States; ^2^ Department of Psychiatry, University of California San Diego, San Diego, CA, United States; ^3^ San Diego State University/University of California San Diego Joint Doctoral Program in Clinical Psychology, San Diego, CA, United States; ^4^ Department of Radiology, University of California San Diego, San Diego, CA, United States; ^5^ Department of Pathology, University of California San Diego, San Diego, CA, United States; ^6^ Department of Neurosurgery, University of California San Diego, San Diego, CA, United States; ^7^ Department of Neurosciences, University of California San Diego, San Diego, CA, United States; ^8^ Department of Radiation Medicine & Applied Sciences, University of California San Diego, San Diego, CA, United States

**Keywords:** memory, fornix, neurosurgery, myxoid glioneuronal tumor, intraventricular, resection

## Abstract

**Background:**

Recently recognized as a distinct entity, a myxoid glioneuronal tumor (MGNT) is a rare, low-grade central nervous system tumor. MGNTs are commonly located at the septum pellucidum or in the third ventricle, increasing the likelihood of tumor or treatment-related damage to adjacent structures critical for memory, such as the fornix. Though there have been a handful of case reports of neurosurgical and oncological outcomes of MGNTs, memory outcomes following resection of MGNTs adjacent to the fornix have not been previously reported.

**Methods:**

We present a case of a high functioning female for whom an MRI revealed an incidental finding of an intraventricular tumor adjacent to the fornix bilaterally. The patient underwent resection of the tumor followed by MRI surveillance without additional oncologic intervention. Due to reported cognitive problems, the patient was referred for serial neuropsychological evaluations.

**Results:**

Post-operative MRI following resection revealed cytotoxic edema followed by selective, progressive atrophy of the bilateral anterior fornices. Post-surgically, the patient developed an isolated verbal memory impairment, which persisted one-year post resection with minimal improvement. The memory impairment impacted the patient’s everyday functioning, including the ability to work in a cognitively demanding job.

**Conclusion:**

This unique case demonstrates the critical role of the bilateral fornix in verbal memory and underscores the importance of a careful risk/benefit analysis when considering neurosurgical intervention to MGNTs and other intracranial lesions adjacent to this structure during neurosurgical planning.

## Introduction

Myxoid glioneuronal tumor (MGNT) is a rare central nervous system tumor, first described in 2018 (1) and newly recognized as its own entity per the 2021 revision of the World Health Organization (WHO) classification (2). MGNTs typically arise at the septum pellucidum, foramen of Monro, or periventricular white matter of the lateral ventricle, placing them adjacent to structures critical for cognition such as the fornix—a C-shaped white matter tract that originates in the hippocampus. MGNTs are slow-growing, grade 1 tumors primarily treated with surgical management, with minimal rates of reoccurrence based on a small number of patients with long-term follow-up (3, 4). There appears to be no benefit of radiation, cytotoxic chemotherapy, or targeted medical therapy. However, proximity to medial structures important for memory and the possible impact on functional outcomes is an important consideration in clinical decision-making and surgical planning. Recently, MGNTs have been characterized in terms of clinical course, imaging, genetic, and histopathologic characteristics (3, 4). However, cognitive and functional outcomes following resection of MGNTs that are impinging on the fornix have not been described.

In humans and animals, the fornix is a critical structure supporting episodic memory (5, 6). Damage to the fornix leads to deficits in spatial navigation and learning in rodents and non-human primates, and to anterograde and retrograde amnesia in humans (5, 7). However, human studies of fornix-associated memory impairment have typically come from patients who have coexisting damage to other diencephalic or medial temporal structures. Isolated fornix lesions are rare, obscuring the unique role of the fornix in healthy memory function and what represents a typical course of recovery following damage to the fornix.

We present a patient diagnosed with an intraventricular MGNT adjacent to the fornix bilaterally who developed a selective verbal memory impairment following an uncomplicated transcallosal resection. Subtotal resection was carried out, with no disruption to the tumor adjacent to the fornices. There was no significant bleeding, and no macroscopic blood vessels were encountered. The patient developed post-operative cytotoxic edema followed by atrophy of the bilateral fornices. Neuropsychological testing demonstrated anterograde verbal memory impairment, which corresponded to the loss of forniceal function. We highlight the role of the fornix in memory and discuss implications for clinical management of similar patients.

## Case description

**Figure 1:**
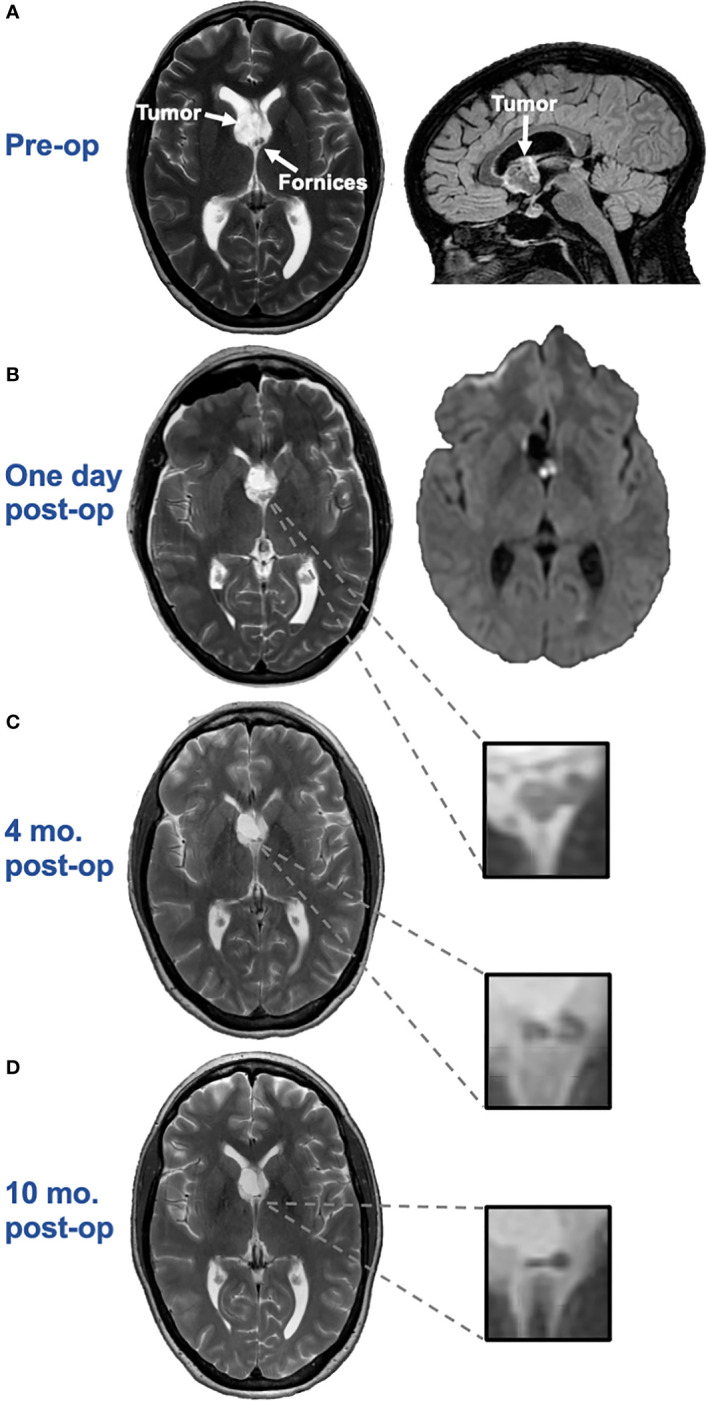
**(A)**. Axial T2 (left) and sagittal 3D CUBE FLAIR (right) images from brain MRI approximately two months before surgery demonstrate an intraventricular mass with a bubbly appearance centered at the right aspect of the septum pellucidum, near the fornices. **(B)**. Axial T2 image (left) and axial diffusion-weighted image (DWI) (right) one day post-surgery demonstrates swelling and restricted diffusion of the bilateral anterior fornices suggesting forniceal injury. The hyperintense signal on the axial DWI had corresponding hypointense signal on the apparent diffusion coefficient (ADC) map, not pictured here, confirming true restricted diffusion. **(C)**. Axial T2 image approximately 4-months after surgery demonstrates irregular and mildly atrophic appearance of the fornices, compatible with evolution of previously seen cytotoxic edema in this region. **(D)**. Axial T2 image approximately 10-months after surgery demonstrates progressive atrophic appearance of the fornices.

An otherwise healthy right-handed female in her 30s with 20 years of education presented to the emergency department after a motor vehicle crash in which she was the driver and was hit head-on by another driver. A full trauma evaluation revealed orthopedic injuries, with no evidence of head injury, loss of consciousness, or focal neurological symptoms. A CT revealed an incidental brain mass, which led to referral for MRI that demonstrated a non-enhancing lesion measuring 2.2 cm centered at the right aspect of the septum pellucidum (**Figure 1A**), with associated mild obstructive hydrocephalus of the lateral ventricles. After consultation with the neurosurgical service and discussion of all treatment options, the patient elected to undergo a right frontal craniotomy for interhemispheric transcallosal subtotal resection of the tumor located within the septum pellucidum. Surgery was carried out with an appreciation of the risk of forniceal injury, with planned subtotal resection leaving a rim of tumor tissue overlying the fornices.

**Figure 2:**
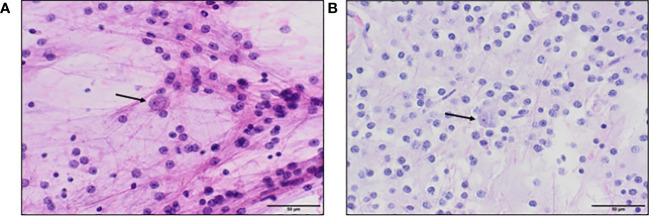
**(A)**. Hematoxylin and eosin (H&E) stained squash preparation and **(B)**. tissue section both show a dual cell population, with a predominance of uniform small round oligodendrocyte-like cells in a fibrillar background, focally accompanied by floating neurons (arrows), which appear larger, with more cytoplasm, and prominent nucleoli.

Histologic examination revealed a low-grade glioneuronal tumor with floating neurons in a faintly basophilic matrix surrounded by bland oligodendrocyte-like tumor cells (**Figure 2**). Immunohistochemical staining revealed the oligodendrocyte-like cells to be diffusely positive for glial fibrillary acidic protein (GFAP). NeuN and synaptophysin stains highlighted scattered floating neurons but were negative in the oligodendrocyte-like cells. A neurofilament stain demonstrated abundant background axonal processes. A Ki-67 index was overall very low, but in select fields approached 5% nuclear staining. An IDH1-R132H stain was negative. Overall, the histologic and immunohistochemical features of this septal tumor were most indicative of MGNT. Molecular analysis was then performed including a next-generation sequencing panel covering 397 genes, as well as chromosomal microarray analysis. Clinically significant alterations detected included a characteristic *PDGFRA* p.K385L mutation (1), confirming the diagnosis of MGNT, as well as very low gains of chromosomes 6, 7, 9, and 10 detected by microarray. No other clinically significant mutations were identified.

There were no unusual operative complications. An MRI one-day post-surgery revealed swelling and restricted diffusion of the bilateral anterior fornices, compatible with cytotoxic edema and raising concern for forniceal injury (**Figure 1B**). An MRI four-months post-resection revealed irregular and mildly atrophic appearance of the fornices, compatible with evolution of the previously seen cytotoxic edema (**Figure 1C**). A repeat MRI 10-months post-resection showed progressive atrophy of the fornices (**Figure 1D**).

The patient did not report any cognitive symptoms prior to the tumor resection and did not experience difficulties at her job as an academic postdoctoral researcher. Following resection, the patient noted new onset short-term memory difficulty. She reported forgetting details of conversations, recent events, whether she had taken her medication or fed her dogs, and difficulty managing appointments, requiring help from family for several months post-surgery. She experienced mental fatigue and difficulty sequencing complex instructions (e.g., lab protocols as part of a highly demanding job). She completed speech therapy and cognitive rehabilitation which helped her to employ compensatory strategies, including using a detailed daily journal and calendar reminders.

The patient completed a neuropsychological evaluation 4- and 12-months post-resection. **Table 1** shows performance on cognitive measures relative to individuals of her age (and education, sex, and race/ethnicity where available). She demonstrated strong performances (i.e., from one to two standard deviations above the mean of cognitively healthy controls) in most domains including attention, processing speed, executive function, language, visuospatial function, visual memory, and motor skills. However, she showed an isolated verbal learning and memory deficit, with performances ranging from 1.5 to 3 standard deviations below the mean of the normative sample. She showed difficulty with encoding, and to a greater degree, with retention and retrieval of learned information during testing, consistent with her described memory problems in everyday life. This pattern of memory impairment persisted one-year post-resection, though she demonstrated some evidence of improvement in retrieval efficiency, likely related to increased utilization of compensatory strategies. Performances in other cognitive domains remained stable or improved from 4- to 12-months post-surgery. The patient reported a reduced quality of life related to her memory impairment, primarily within the domains of her social and work life. Her memory impairment greatly interfered with her ability to learn, recall, and apply new information and sequence protocols in the context of a highly cognitively demanding job.

**Table 1:** Summary of neuropsychological testing completed 4-months and 1-year after surgery.

Cognitive Domain	Test	4 months post-op			1-year post-op		
		Raw	*T*	%ile	Raw	*T*	%ile
Verbal Memory	CVLT-3 Trials 1-5	37	**32**	4	41	**38**	13
	CVLT-3 SDFR	6	**33**	5	7	**34**	6
	CVLT-3 LDFR	4	**24**	1	7	**31**	3
	CVLT-3 Recognition Discrim.	2.3	**36**	8	1.9	**34**	6
	CVLT-3 Intrusions	25	**20**	<1	11	**34**	6
	WAIS-IV Logical Memory I	20	**36**	8	23	**39**	14
	WAIS-IV Logical Memory II	1	**15**	<1	11	**28**	2
	WAIS-IV Logical Memory Recognition	24	—	26-50	26	—	51-75
Visual Memory	BVMT Learning	33	63	91	29	55	70
	BVMT Delayed Recall	11	55	70	10	49	47
	BVMT Recognition	6	—	>16	6	—	>16
	Rey-Osterrieth Complex Figure Test: 30-minute delay	—	—	—	20	54	67
Language	Boston Naming Test	58	53	63	—	—	—
	Category Fluency	40	50	50	45	57	75
Attention/Processing Speed	WAIS-IV Digit Span	41	70	98	41	70	98
	WAIS-IV Coding	76	41	18	89	47	37
	WAIS-IV Symbol Search	39	51	54	48	66	95
Executive Function	DKEFS Letter Fluency	56	67	95	64	77	>99
	DKEFS CWIT Inhibition	44	57	75	43	57	75
	DKEFS Number-letter Switching	53	57	75	31	63	91
	WCST Perseverative Errors	4	47	37	—	—	—
Visuospatial	BVMT Copy	12	—	—	—	—	—
	WAIS-IV Block Design	62	65	94	—	—	—
Motor	Grooved Pegboard - dominant	50	60	84	—	—	—
	Grooved Pegboard - nondominant	55	60	84	—	—	—

Bolded are scores that are greater than 1 standard deviation below the normative sample mean (i.e., a T-score < 40); T-scores have a mean of 50 and a standard deviation of 10; %ile, percentile; CVLT-3, California Verbal Learning Test-Third Edition (normed for age, sex, and education); WAIS-IV, Wechsler Adult Intelligence Scale-4^th^ Edition (normed for age, sex, education, and ethnicity); WMS-IV, Weschler Memory Scale-4^th^ Edition (normed for age, sex, education, and ethnicity; BVMT, Brief Visuospatial Memory Test-Revised (normed for age); DKEFS, Delis-Kaplan Executive Function System (normed for age). Boston Naming Test and Grooved Pegboard scores are normed for age, sex, education, and ethnicity; the Rey-Osterrieth Complex Figure Test is normed for age.

## Discussion

We present the unique case of a young high functioning woman who suffered a new-onset verbal memory impairment following transcallosal resection of an intraventricular MGNT that impinged upon the fornix bilaterally. Notably, the memory impairment persisted one-year following surgery. This serves as strong evidence that isolated damage to the bilateral anterior fornices can produce a selective and sustained memory impairment similar to hippocampal lesions (5). This is a critical point given the important role of the fornices in memory encoding and consolidation. In addition, this novel case demonstrates that anterior midline structures, including the fornices, can be injured even when care is taken to avoid direct trauma during resection of an adjacent MGNT.

**Figure 3:**
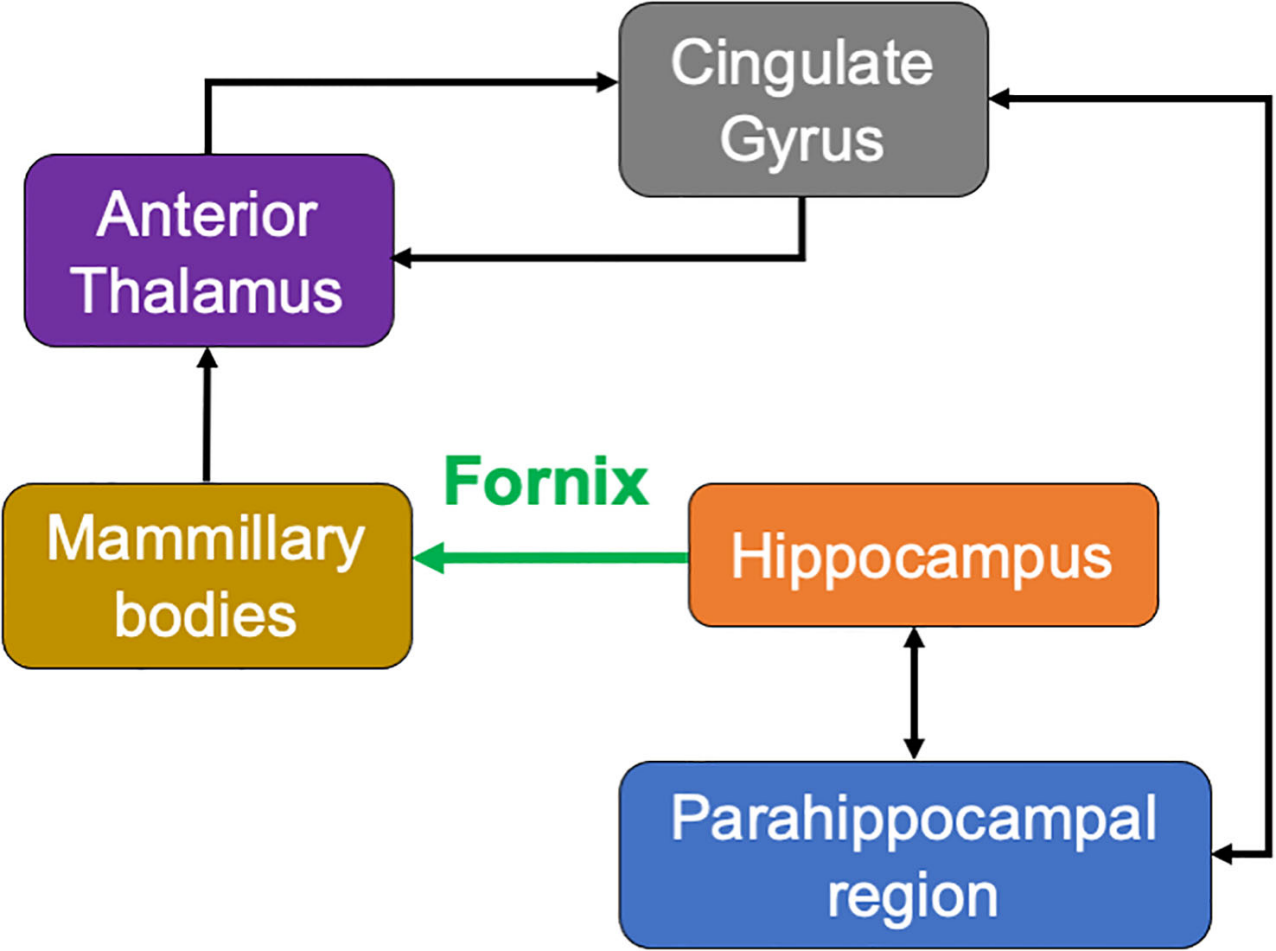
Schematic of the classic Papez circuit—a medial limbic network important for episodic memory. Information from cortical association areas is sent to the hippocampus *via* the cingulate gyrus, parahippocampal gyrus/entorhinal cortex, and cingulum bundle (white matter fibers). Processed information from the hippocampus is sent *via* the fornix (green) to the mammillary bodies of the hypothalamus. Information from the mammillary bodies is sent to the anterior nuclei of the thalamus *via* the mammillothalamic tract, which then projects fibers to the cingulate gyrus, parahippocampal gyrus/entorhinal cortex, and cingulum back to the hippocampus.

The fornix is the main white matter output tract of the hippocampus with projection to the mammillary bodies. It is a critical part of the Papez circuit interconnecting multiple memory interfaces including the medial temporal lobe, the medial diencephalon, and the basal forebrain (**Figure 3**). Damage to midline structures including the fornix, mamillary bodies, and thalamus can result in diencephalic amnesia—a severe form of anterograde amnesia. However, diencephalic amnesia has been classically studied in patients with *diffuse* damage, such as thiamine-deficient Korsakoff syndrome (8) or widespread lesions from other etiologies. Anterograde memory impairment can also follow colloid cyst resections (7, 9, 10), a closer analogue to the present case as colloid cysts are benign, centered at the foramen of Monro, and may require a midline surgical approach through the corpus callosum. However, here too, in addition to the fornix almost all reported cases of uncomplicated colloid cyst resections had collateral damage to other structures including the mamillary bodies (9, 11), hippocampus (11), or basal forebrain (11). This makes it difficult to ascertain the unique role of the fornix in memory. In contrast, the fornix lesion in our patient was not accompanied by any apparent damage to these structures, thus providing compelling evidence of the essential role of the bilateral anterior fornix in verbal memory. Notably, there is considerable variability in the degree of fornix-associated memory loss previously reported, from irreversible amnesia (11) to partial resolution over a year in monkeys (12) and humans (10), with variability in outcome likely related to the spatial location, size, and laterality of the fornix lesion, memory construct evaluated, and surgical approach. However, fornix lesions that most consistently led to severe and sustained memory impairment appear to be of the bilateral anterior columns (6, 11), as is the case for the current patient.

## Limitations and future directions

Non-invasive imaging tools such as diffusion-weighted MRI allow us to study the microstructural white matter integrity or connectivity of the fornix *in-vivo.* However, reconstructing the fornix requires collection of many diffusion directions (e.g., >30) given its narrow size, sharp bending angle, and close proximity to the ventricles leading to a high susceptibility to partial voluming and effects of crossing fibers (5). As our diffusion-weighted imaging sequence was collected as part of a standard clinical protocol, it did not contain enough diffusion-encoding directions to carry out additional more sophisticated tractography analyses that would somewhat mitigate these concerns. Advanced sequences with high angular resolution diffusion imaging (HARDI) models are better suited to reliably reconstruct the fornix and evaluate its contribution to episodic memory and inform risk assessment in surgical planning.

One potential future direction for group-level studies is to consider information beyond the anatomical boundaries of the lesion site by examining maps of lesion-induced impairments to brain *networks*. This could be accomplished using lesion network mapping (13), allowing for an estimation of the impact of any potential lesion to the fornix in disruption of a wider network of structures which may be functionally and structurally connected to the fornix, such as the diencephalic and medial temporal structures illustrated in **Figure 3**. However, a noteworthy limitation of this method is difficulty determining which of the identified regions of the whole-brain network are *causally* related to behavior (14).

## Conclusion

Though considered low-grade tumors, the clinical decision tree for intraventricular tumors presents several challenging medical decision points. While an experienced neuro-radiologist can provide a differential diagnosis of the lesion based on radiographic appearance, a pathological sample is required for definite diagnosis. Although surveillance without intervention of a presumed low-grade lesion presents minimal risk for progressive cognitive decline, MGNTs and other intraventricular tumors are associated with risk of obstructive hydrocephalus, headaches, confusion, lethargy, seizures, and even death (4). Conversely, surgical resection may facilitate lesion diagnosis, improve symptom control, decrease risk of recurrence, and improve survival and quality of life. However, as documented here, surgery involves a risk to memory when the tumor is situated near structures critical for cognition such as the fornix and diencephalon, which when damaged can lead to memory impairment (5). In this case, given that care was taken to avoid direct forniceal injury, the observed injury was possibly related to vascular compromise resulting from injury to the end arterial branches arising from the anterior cerebral arteries and traversing the tumor substance. The literature on fornix lesions and memory generally suggests that *bilateral anterior* lesions appear to lead to more severe memory impairment (6), though unilateral lesions may also cause amnesia (15). Therefore, additional consideration can be given to exact tumor location when aiming for a maximal safe resection. A trade-off analysis between risk to cognitive ability and risk to health is imperative when presented with an intraventricular MGNT.

## Data availability statement

The datasets presented in this article are not readily available because this data in part comes from a clinical evaluation and is not de-identified. Requests to access the datasets should be directed to Alena Stasenko; astasenko@ucsd.edu.

## Ethics statement

The requirement of ethical approval was waived by University of California, San Diego IRB for the studies involving humans because University of California, San Diego IRB. The studies were conducted in accordance with the local legislation and institutional requirements. The participants provided their written informed consent to participate in this study. Written informed consent was obtained from the individual(s) for the publication of any potentially identifiable images or data included in this article.

## Author contributions

AS: Conceptualization, Data curation, Writing – original draft. EK: Conceptualization, Writing – review & editing. JR: Conceptualization, Formal Analysis, Visualization, Writing – review & editing. JK: Data curation, Writing – review & editing. NF: Conceptualization, Data curation, Visualization, Writing – review & editing. VG: Data curation, Visualization, Writing – review & editing. MS: Conceptualization, Writing – review & editing. JS: Conceptualization, Writing – review & editing. CM: Conceptualization, Supervision, Writing – review & editing.
